# Respiratory compromise in children presenting to an urban emergency department of a tertiary hospital in Tanzania: a descriptive cohort study

**DOI:** 10.1186/s12873-019-0235-4

**Published:** 2019-02-28

**Authors:** Biita Muhanuzi, Hendry R. Sawe, Said S. Kilindimo, Juma A. Mfinanga, Ellen J. Weber

**Affiliations:** 1 Emergency Medicine Department, Muhimbili University of Health and Allied Sciences, P.O. Box 65001, Dar es Salaam, Tanzania; 2 Emergency Medicine Department, Muhimbili National Hospital, Dar es Salaam, Tanzania; 3 Department of Emergency Medicine, University of California, San Francisco, CA USA

**Keywords:** Respiratory compromise, Emergency medicine, Tanzania, Respiratory distress, Paediatrics, Emergency care

## Abstract

**Background:**

Respiratory compromise is the leading cause of cardiac arrest and death among paediatric patients. Emergency medicine is a new field in low-income countries (LICs); the presentation, treatment and outcomes of paediatric patients with respiratory compromise is not well studied. We describe the clinical epidemiology, management and outcomes of paediatric patients with respiratory compromise presenting to the first full-capacity Emergency Department in Tanzania.

**Methods:**

This was a prospective cohort study of paediatric patients (< 18 years) with respiratory compromise (respiratory distress, respiratory failure or respiratory arrest) presenting to the Emergency Medicine Department of Muhimibili National Hospital (EMD-MNH) in Dar es Salaam, from July–November 2017. A standardized case report form was used to record demographics, presenting clinical characteristics, management and outcomes. Primary outcomes were hospital mortality and secondary outcomes were EMD mortality, 24-h mortality, incidence of cardiac arrest in the EMD, length of stay, ICU admission, and risk factors for mortality.

**Results:**

We enrolled 165 children; their median age was 12 months [IQR: 4–36 months], and 90 (54.4%) were male. At presentation 92 (55.8%) children were in respiratory failure. Oxygen therapy was initiated for 143 (86.7%) children, among which 21 (14.7%) were intubated.

The most common aetiologies were pneumonia followed by congenital heart disease and sepsis. The majority 147 (89.1%) of children were admitted to the hospital, with 20 (12%) going to ICU. Four (2%) children were discharged from EMD and 14 (8.5%) died in the EMD. In the EMD, 18 children developed cardiac arrest, with two surviving to hospital discharge. Overall 51 (30.9%) children died; 84% of deaths were in children under five years. Risk of mortality was increased in children presenting with decreased consciousness (RR = 2.2 (1.4–3.4)), hypoxia RR = 2.6 (1.6–4.4)) or bradypnoea (RR = 3.9 (2.9–5.0)), and those who received CPR (RR = 3.7 (2.7–5.2)) and intubation (RR = 3.1 (2.1–4.5)).

**Conclusions:**

In this EMD of a LICs, respiratory compromise in children carries high mortality, with children of young age being the most vulnerable. Many children arrived in respiratory failure and few children received ICU care. Outcomes can be improved by earlier recognition to prevent cardiac arrest, and more intensive treatment, including ICU and assisted ventilation.

## Background

Respiratory compromise is the leading cause of death worldwide, especially in children below the age of 5 years [1, 2]. Most of these deaths occur in low-income countries (LICs). Respiratory compromise is more common in children than in adults because of anatomical and physiological differences like small airway, immature immune system and under developed compensatory mechanisms.

Respiratory compromise is a continuum of respiratory distress, respiratory failure and respiratory arrest. Respiratory failure refers to failure of oxygenation or ventilation or both and respiratory distress refers to increased work of breathing [3]. Early identification, early intervention and close monitoring can mitigate against poor outcomes [4–6]. Such interventions are best provided in an emergency department where practitioners are trained in the management of such patients [7].

Tanzania health system consists of dispensaries at village level, health centres at ward level, district hospital, regional hospital, zonal hospital and national hospital. Patients including children are referred from low-level facility to higher-level facility. In Tanzania there is no children hospital, so Muhimbili National Hospital receives both adult and children from all regional hospitals and some directly from home and from private hospitals [8].

Tanzania opened its first full capacity emergency department in 2010 and began a residency-training program at that time. Current emergency medicine practice in LICs is based on research primarily conducted in high-income countries, where the aetiology, severity and resources available to treat patients with respiratory compromise are very different from LICs. Despite the ongoing advancement in the development of the emergency care infrastructure in Tanzania [9], there is insufficient data to guide development of specific clinical guidelines for training, management and resources required to treat paediatric patients with respiratory compromise in acute care settings in limited income settings [10].

To address the paucity of data, we aimed to describe the clinical epidemiology, management and outcomes of paediatric patients with respiratory compromise presenting to the first full capacity emergency medicine department in Tanzania.

## Methods

### Study design

This was a prospective cohort study of paediatric patients aged from 1 month to less than 18 years with respiratory compromise, presenting to the Emergency Medicine Department of Muhimbili National Hospital (EMD-MNH) from July 2017 to November 2017.

### Study setting

Muhimbili National Hospital is a tertiary hospital located in Dar es Salaam, Tanzania. It has a bed capacity of 1500 with around 1000 to 1200 inpatient admissions per week. The EMD is part of the MNH and it is the point of entry to the hospital for most patients [11].

EMD-MNH opened in 2010, making it the first public full capacity emergency department in the country. It operates 24 h a day and sees 150–200 patients per day; approximately one quarter of them are children under 18 years. As MNH is a tertiary referral hospital, many of the patients attending the EMD are transferred from regional or district hospitals where they may have received some previous treatment.

The EMD is staffed with emergency physicians, residents in an emergency medicine program, medical officers and critical care nurses. Acutely ill children at MNH-EMD receive resuscitation and diagnostic workup before being disposed to the appropriate ward, which may include Acute Paediatric Care Unit (APCU) or the adult ICU. A paediatric specialist on call reviews paediatric patients with respiratory compromise who may need ICU, APCU admission or major surgical interventions before the patients leave the EMD.

### Study protocol

Patients were enrolled 12 h a day during the day shift (7,30 am to 7:30 pm) Monday through Saturday. Paediatric patients presenting to the EMD with respiratory distress, respiratory failure or respiratory arrest as defined by American Heart Association were eligible for the study [3]. Paediatric Early Warning Sign parameters were used as a reference for tachypnoea, bradypnoea, tachycardia and bradycardia in different age groups [12]. For the children who met inclusion criteria, the parent/guardian provided signed informed consent. Demographics, clinical presentation, EMD management, EMD diagnosis, and EMD outcomes of all enrolled children were collected from the EMD physician caring for the patient and review of the patients’ electronic file. All children admitted to the hospital were followed up until discharge or death. Outcomes were recorded at time of disposition from the EMD, at 24-h post EMD disposition, and on the day of discharge from hospital (or death).

Primary outcomes were hospital mortality and secondary outcomes were EMD mortality, 24-h mortality, and incidence of cardiac arrest in the EMD, length of stay, ICU admission and risk factors for mortality.

### Data analysis

Data from REDCap (Version 7.2.2, Vanderbilt, Nashville, TN, USA) were exported to Statistical Package for Social Science (SPSS version 22.0, IBM Corp., Armonk, NY, USA) for analysis. For the primary outcomes, descriptive statistics; median, proportion, interquartile range [IQR] and counts, are displayed. For the secondary outcome of risk factors for mortality, we used relative risk.

## Results

**Fig. 1:**
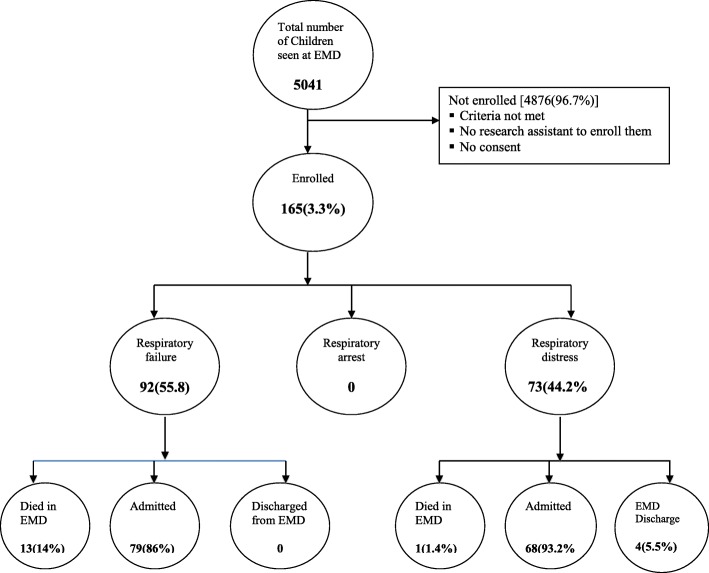
Flow chart of pediatric patients presenting with respiratory compromise in Emergency Medicine Department

During the enrolment times, 165 children with respiratory compromise attended the EMD and all consented to be in the study. Among them 92 (55.8%) presented in respiratory failure, 73 (44.2%) in respiratory distress and no children had respiratory arrest during enrolment (Fig. 1).

### Patient characteristics

**The** median age was 12 months (IQR 4–36 months), and slightly more children were male. 69% had been referred from another hospital; 48.5% had existing comorbidities. Presenting complaints included; difficulty in breathing 153(92.7%), fever 89(53.9%) and cough 73(44.2%) The most common signs of respiratory failure were hypoxia 79(85.9%) on room air (SPO2 < 91%) followed by decreased level of consciousness 42(45.6%) (Table 1).

**Table 1:** Gender, age, referral status, comorbidities and presenting complaints

Variable	*N* = 165
	n (%)
Male	90 (54.5)
0- < 1 years	83 (50.3)
1- < 5 years	49 (29.7)
5- < 10 years	10 (6.1)
10- < 18 years	23 (13.9)
Referred	114 (69.1)
Co-Morbidities	
Congenital heart disease (CHD)	33 (20)
Cerebral Palsy (CP)	10 (6.1)
Human Immunodeficiency Virus (HIV)	8 (4.8)
Other	29 (17.6)
Clinical presentation	
Difficulty inbreathing	153 (92.7)
Fever	89 (53.9)
Cough	73 (44.2)
Decreased level of consciousness	42 (25.5)
Convulsions	18 (10.9)
Hypoxia (SPO2 < 91% on room air)	79 (47.9)
Tachycardia*	59 (35.8)
Tachypnea*	54 (32.7)
Bradypnoea*	11 (23.6)
Bradycardia *	9 (5.5)
Temperature < 35 or > 37.8 C	39 (23.6)
Dianoses**	
Pneumonia	90 (54.5)
Sepsis	52 (31.5)
Congenital heart disease	48 (29.1)
Severe Anemia	13 (7.9)
Severe malaria	12 (7.3)

*According to age, ** A child can have more than one diagnoses

### Investigations performed in EMD

Point of care random blood glucose was assessed in all children enrolled in the study and 16 (9.7%) of them had hypoglycemia of less than 3 mmol/L (Table 2). Malaria rapid diagnostic test was the second most commonly ordered test in 117 (70.9%) of children and 6.8% of tests were positive (Table 2). Blood gas analysis was performed in 90 (54.5%) of children and 71% of these were abnormal **(**Table 2**).** Chest radiography was done in 61(37%) children and 29.5% were abnormal (Table 2). Urgent blood grouping and typing was performed in 19 (11.5%) children. Serum lactate was raised in 41(56.9%) of all of the children tested for serum lactate (Table 2).

**Table 2:** EMD diagnostic tests performed among children with respiratory compromise

Tests perfomed in emd	Patients receiving test (*n* = 165)	Abnormal test results
	*n* (%)	*n* (%)
Random blood glucose	165 (100)	16 (9.7)
Malaria rapid diagnostic test	117 (70.9)	8 (6.8)
Complete blood count	96 (58.2)	42 (43.8)
Blood gases analysis	90 (54.5)	64 (71.1)
Serum electrolytes	78 (47.3)	20 (25.6)
Serum lactate	72 (43.6)	41 (56.9)
Point of care hemoglobin level	68 (41.2)	60 (88.2)
Blood slide for malaria	31 (18.8)	8 (25.8)
Chest x-ray	61 (37.0)	18 (29.5)

### EMD treatment

Common treatments initiated in EMD were oxygen therapy 143 (86.7%), antibiotics 119 (72.1%) and intravenous fluids 99 (60%). Blood was transfused in 15 of the 19 patients who had a typing. CPR was given to 18 (10.9%) children after cardiac arrest in the EMD (Table 3). Children received intubation were 18; of these 14 were intubated during resuscitation for cardiac arrest (Table 3).

**Table 3:** Frequency of Interventions initiated in the EMD

Emd interventions	*N* = 165
	*n* (%)
Oxygen therapy	143 (86.7)
Antibiotics	119 (72.1)
Intravenous fluids	99 (60.0)
Bronchodilators /nebulization	25 (15.2)
Electrolyte replacement	20 (12.1)
Nasogastric tube insertion	19 (11.5)
Suctioning	18 (10.9)
Endotracheal intubation	18 (10.9)
Cardiopulmonary resuscitation	18 (10.9)
Airway adjunct insertion	16 (9.7)
Blood transfusion	15 (9.1)
Inotropes	14 (8.4)

### EMD impression

The most common EMD impression was pneumonia in 54.5% of children. Sepsis (31.5%) and congenital heart disease (29.1%) were the next most common diagnoses (Table 4).

**Table 4:** EMD outcomes and in hospital Outcomes by age

Outcome	TOTAL N = 165 n (%)	< 5 years *N* = 132 n (%)	≥ 5 years *N* = 33 n (%)
Admitted to general ward	127 (77.0)	104 (78.8)	23 (69.7)
Admitted to ICU/APCU	20 (12.1)	17 (12.9)	3 (9.1)
Discharged from EMD	04 (2.4)	2 (1.5)	2 (6.1)
Died in EMD	14 (8.5)	12 (9.1)	2 (6.1)
Died within 24 h after EMD disposition	09 (5.5)	8 (6.1)	1 (3.0)
Inpatient mortality	37 (22.4)	31 (23.5)	6 (18.2)
Overall mortality	51 (30.9)	43 (32.6)	8 (24.2)

### Outcomes

#### Mortality

Overall hospital mortality was 30.9% (95% CI = 23.85–37.95) **(**Table 4**).** The median age of patients who died was 8 months (IQR = 4–24 months) **(**Table 4**).** There was a greater percentage of deaths among children < 5 compared with older children **(**Table 4**).** Among 14 children who died in the EMD, 13(92.1%) presented with respiratory failure and 1(7.1%) had respiratory distress on arrival **(**Table 4**)**. All four discharged children had respiratory distress **(**Fig. 1**)**. The leading cause of death was pneumonia followed by sepsis.

#### Length of stay

The median length of stay in the EMD was 5 h (range1–27 h), median length of hospital stay was 8 days (range 1–55 days). Among those children who died, the median length of stay before death in EMD was 6 h (IQR = 2.3–8.5 h) and in the wards was 3 days (IQR = 1–4 days).

#### Predictors of mortality

In unadjusted analyses, presentation with decreased consciousness, hypoxia on room air, or bradypnoea were associated with an increased risk of death. Need for an airway adjuvant, or for intubation in the EMD was also associated with death **(**Table 5**).**

**Table 5:** Presenting complaints, abnormal laboratory results, EMD interventions and outcomes

Complaints	Overall n/N	Died *N* = 51 *n* (%)	Survived *N* = 114 *n* (%)	RR (95%CI)
Difficulty inbreathing	153/165	40 (78.4)	113 (99.1)	0.3 (0.2–0.4)
Fever	89/165	24 (47.1)	65 (57.0)	0.8 (0.5–1.2)
Cough	73/165	13 (25.5)	60 (52.6)	0.4 (0.2–0.7)
Decreased consciousness	42/165	22 (43.1)	20 (17.5)	2.2 (1.4–3.4)
Convulsions	18/165	8 (15.7)	10 (8.8)	1.6 (0.9–2.8)
SPO2 < 91%	79/165	36 (70.6)	43 (37.7)	2.6 (1.6–4.4)
Bradypnoea	11/165	11 (21.6)	0	3.9 (2.9–5.0)
Abnormal EMD results				
RBG < 3 mmol/L	16/165	7 (13.7)	9 (7.9)	1.5 (0.8–2.7)
Hemoglobin < 7 g/dl	22/124	8 (15.7)	14 (12.3)	1.2 (0.7–2.2)
WBC (> 11 K/uL)	42/96	8 (15.7)	34 (29.8)	0.5 (0.3–1.1)
Malaria positive	008/117	2 (3.9)	6 (5.3)	0.8 (0.2–2.7)
EMD Interventions				
Oxygen therapy	143/165	41 (80.4)	102 (89.5)	0.6 (0.4–1.1)
Antibiotics	119/165	29 (56.9)	70 (61.4)	0.9 (0.6–1.4)
Nebulization	25/165	7 (13.7)	18 (15.8)	0.9 (0.5–1.7)
Nasogastric tube insertion	19/165	15 (29.4)	4 (3.5)	0.9 (0.4–2.0)
Intubation	18/165	14 (27.5)	4 (3.5)	3.1 (2.1–4.5)
CPR	18/165	16 (31.4)	2 (1.8)	3.7 (2.7–5.2)
Airway adjunct insertion	16/165	16 (31.4)	0	4.8 (3.2–5.7)
Blood transfusion	15/165	6 (17.8)	9 (7.9)	0.1 (0.01–2.3)

*CI = Confidence interval. CPR = Cardiopulmonary resuscitation. RR = Relative Risk*

## Discussion

Globally, respiratory compromise in the paediatric population is the leading cause of emergency visits, cardiac arrest, ICU admission and prolonged hospital stay [5, 10, 12]. In Tanzania the burden, clinical epidemiology, management and outcomes of acute respiratory compromise among paediatric population attending acute care settings has not been previously documented.

In our study, the majority of children with respiratory compromise were below age 5 years, more than half had respiratory failure on arrival and one third died before hospital discharge. The median age of children who died was 8 months, suggesting that those under one year are a particularly vulnerable group. Nearly half of the children enrolled had comorbidities, the most common being congenital heart disease. Pneumonia was the most frequent diagnosis. Notably, the majority of children presented in respiratory failure, and only 12% received ICU care. Risk of death was higher among children who presented in emergency department with hypoxia, decreased level of consciousness, bradypnoea and those who received cardiopulmonary resuscitation, intubation, and airway adjunct insertion in EMD. The risk of death among intubated children was high, because most children were intubated after developing cardiac arrest, and thus there was a delay in EMD in initiating assisted ventilation.

Similar findings regarding the young age of paediatric patients presenting with respiratory compromise have been reported in India, China, Zambia, and Nigeria [5, 13–15]. Children under five years are more likely to develop respiratory compromise than other age groups because of their small airway, underdeveloped central nervous system, less energy store and other anatomical differences compared with older children [16]. They also have higher risk of Respiratory Syncytial Virus infection and this is particularly true of those below one year of age [17].

In our study, the proportion of children arriving with respiratory failure was higher than reported in studies in India and Canada [14, 18]. This finding is likely a result of delayed presentation or delayed transfer to our hospital, which has been documented for other illnesses in LICs.

The leading cause of respiratory compromise was pneumonia, then congenital heart disease and sepsis but the leading causes of death were pneumonia followed by sepsis and congenital heart disease. This is consistent with previous studies in LICs; in Nigeria, Zambia and Malawi, pneumonia was the leading cause of death [6, 14, 19]. A study from India also reported pneumonia as the leading cause of death [14].

In this study, we found that the use of investigations varied. For example, while almost all children had random blood glucose and malaria testing, blood gas analysis was performed in only about half of paediatric patients with respiratory compromise. In a study conducted in India among children with acute respiratory failure, blood gas analysis was performed in all paediatric patients [14]. Blood gases were not performed in our department either due to the cost to the patient or intermittent availability of reagent; a previous study in Ghana described similar barriers to testing [6]. Sawe et al. in 2011 reported unavailability of blood gas analyzer machines in all main referral hospital ICUs in Tanzania [20]. The lower frequency of blood gas analysis seen in this study demonstrates that relying on such investigations is not always practical in low income countries and thus emergency care providers must depend on clinical features to diagnose respiratory failure.

Perhaps surprisingly, chest radiography was performed in only one third of the children. Clinicians in our department may not order chest radiography probably because of the poor quality of portable chest radiography in our hospital, and children were too sick (87% were on oxygen therapy) to go to the radiology department for imaging. There is also an increasing use of point of care lung ultrasound in our department.

Most children received oxygen therapy**;** however, only 11% received mechanical ventilation. In a study in India with a comparable group of patients, mechanical ventilation was required in 33.9% of children with respiratory failure in the emergency department [14]. This suggests that there are insufficient resources in our setting. At the time of this study, MNH had no paediatric ICU, children who require intensive care are admitted to the adult medical ICU which had only 13 bed capacity and no ventilators which can support children of young ages [18]. In Canada and India all paediatric patients with respiratory failure were admitted to ICU from emergency department [14, 18]. Notably, the overall mortality was two times higher in this study than in the study in India.

The high mortality rate of our population can be attributed to several potential reasons. The majority of children arrived in EMD after developing respiratory failure and thus had less reserve despite attempts at lifesaving intervention. The risk factors for mortality reflect this end-stage disease; decreased consciousness, hypoxia and bradypnoea, cardiac arrest, and the need for ventilation. A second reason may be failure of EMD physicians to initiate invasive measures sooner; the median length of stay for children who developed cardiac arrest in our department was 6 h, suggesting that there may have been opportunity to intervene sooner if respiratory failure had been recognised. However, another reason for delayed intervention may be the lack of ventilators in the EMD and in hospital main ICU. Finally, the high mortality is likely attributable to the low ICU admission rate resulting from a lack of a paediatric ICU and less ICU bed capacity in the adult ICU [18, 20]. Another possibility could be poor adherence to respiratory management guidelines or poor quality of interventions.

### Limitations

This was a single site descriptive study with a small sample conducted over a relatively short duration, limiting our ability to account for seasonal variation. Patient enrolment was only performed for 12 h a day and it is possible that the types of patients arriving at night are different. Hence our results may not necessarily be fully representative of our population, or generalizable to other emergency departments with different patient populations in different seasons. However, as noted above, our findings regarding demographics, and etiologies are consistent with prior studies and our higher mortality rate can be explained by the differences in our health care system and setting.

## Conclusions

Paediatric patients presenting to our EMD with respiratory compromise had a high mortality rate. This is likely due to a combination of late presentation, in which the majority was in respiratory failure, underlying comorbidities, and limitations on resources. This information suggests that efforts should be placed on early recognition and referral, developing standard investigation and resuscitation algorithms for the EMD, and allocating additional resources for critical care.

## Abbreviations

ABG: Arterial Blood Gases
APCU: Acute pediatric care unit
AVPU: Alert-Verbal-Pain-Unresponsive
CHD: Congenital Heart Disease
ECG: Electrocardiogram
EMD: Emergency Medicine Department
ICU: Intensive Care Unit
LAMA: Leaving Against Medical Advice
MNH: Muhimbili National Hospital
MUHAS: Muhimbili University of Health and Allied Sciences
PALS: Paediatric Advanced Life support
PEWS: Paediatric Early Warning Score
RC: Respiratory Compromise
RCI: Respiratory Compromise Institute

## References

[1] Roomaney RA, Pillay-van Wyk V, Awotiwon OF, Dhansay A, Groenewald P, Joubert JD, et al. Epidemiology of lower respiratory infection and pneumonia in South Africa (1997–2015): a systematic review protocol. BMJ Open. 2016 15;6(9):e012154. 10.1136/bmjopen-2016-012154 PMC5030548 27633638

[2] University of Washington, Institute for Health Metrics and Evaluation. The global burden of disease: generating evidence, guiding policy [Internet]. 2013 [cited 2019 Feb 7]. Available from: http://www.healthmetricsandevaluation.org/sites/default/files/policy_report/2011/GBD_Generating%20Evidence_Guiding%20Policy%20FINAL.pdf

[3] Chameides L, Ralston M, American Academy of Pediatrics, American Heart Association. Pediatric advanced life support [Internet]. Dallas, TX: American Heart Association; 2011 [cited 2019 Feb 7]. Available from: https://archive.org/details/pediatricadvance00amer

[4] Krauss BS, Harakal T, Fleisher GR (1991). The spectrum and frequency of illness presenting to a pediatric emergency department. Pediatr Emerg Care.

[5] Nitu ME, Eigen H. Respiratory failure. Pediatr Rev 2009;30(12):470–477; quiz 478. 10.1542/pir.30-12-470 19952128

[6] Japiong KB, Asiamah G, Owusu-Dabo E, Donkor P, Stewart B, Ebel BE (2016). Availability of resources for emergency care at a second-level hospital in Ghana: a mixed methods assessment. Afr J Emerg Med.

[7] Sawe HR, Mfinanga JA, Mwafongo V, Reynolds TA, Runyon MS (2015). Trends in mortality associated with opening of a full-capacity public emergency department at the main tertiary-level hospital in Tanzania. Int J Emerg Med.

[8] Simba DO, Mbembati NAA, Museru LM, Lema LEK (2008). Referral pattern of patients received at the national referral hospital: challenges in low income countries. East Afr J Public Health.

[9] Shayo EH, Senkoro KP, Momburi R, Olsen ØE, Byskov J, Makundi EA (2016). Access and utilisation of healthcare services in rural Tanzania: a comparison of public and non-public facilities using quality, equity, and trust dimensions. Glob Public Health.

[10] Reynolds TA, Mfinanga JA, Sawe HR, Runyon MS, Mwafongo V (2012). Emergency care capacity in Africa: a clinical and educational initiative in Tanzania. J Public Health Policy.

[11] Reynolds T, Sawe HR, Lobue N, Mwafongo V (2012). Most frequent adult and pediatric diagnoses among 60,000 patients seen in a new urban emergency Department in Dar Es Salaam. Tanzania Ann Emerg Med.

[12] Lambert V, Matthews A, MacDonell R, Fitzsimons J. Paediatric early warning systems for detecting and responding to clinical deterioration in children: a systematic review. BMJ Open [Internet]. 2017 Mar 10 [cited 2019 Feb 7];7(3). Available from: https://www.ncbi.nlm.nih.gov/pmc/articles/PMC5353324/10.1136/bmjopen-2016-014497 PMC5353324 28289051

[13] Tazinya AA, Halle-Ekane GE, Mbuagbaw LT, Abanda M, Atashili J, Obama MT. Risk factors for acute respiratory infections in children under five years attending the Bamenda Regional Hospital in Cameroon. BMC Pulm Med [Internet]. 2018 Jan 16 [cited 2019 Feb 7];18. Available from: https://www.ncbi.nlm.nih.gov/pmc/articles/PMC5771025/10.1186/s12890-018-0579-7 PMC5771025 29338717

[14] Singh V (2005). The burden of pneumonia in children: an Asian perspective. Paediatr Respir Rev.

[15] Oguonu T, Adaeze Ayuk C, Edelu BO, Ndu IK. Pattern of respiratory diseases in children presenting to the paediatric emergency unit of the University of Nigeria Teaching Hospital, Enugu: a case series report. BMC Pulm Med. 2014 Jun 10;14:101. 10.1186/1471-2466-14-101 PMC4088915 24916799

[16] Simoes EAF, Cherian T, Chow J, Shahid-Salles SA, Laxminarayan R, John TJ. Acute Respiratory Infections in Children. In: Jamison DT, Breman JG, Measham AR, Alleyne G, Claeson M, Evans DB, et al., editors. Disease Control Priorities in Developing Countries [Internet]. 2nd ed. Washington (DC): World Bank; 2006 [cited 2019 Feb 7]. Available from: http://www.ncbi.nlm.nih.gov/books/NBK11786/

[17] Trachsel D, McCrindle BW, Nakagawa S, Bohn D (2005). Oxygenation index predicts outcome in children with acute hypoxemic respiratory failure. Am J Respir Crit Care Med.

[18] Schindler MB, Bohn D, Cox PN, McCrindle BW, Jarvis A, Edmonds J (1996). Outcome of out-of-hospital cardiac or respiratory arrest in children. N Engl J Med.

[19] Bates M, Shibemba A, Mudenda V, Chimoga C, Tembo J, Kabwe M (2016). Burden of respiratory tract infections at post mortem in Zambian children. BMC Med.

[20] Sawe HR, Mfinanga JA, Lidenge SJ, Mpondo BC, Msangi S, Lugazia E (2014). Disease patterns and clinical outcomes of patients admitted in intensive care units of tertiary referral hospitals of Tanzania. BMC Int Health Hum Rights.

